# Basic life support education in secondary schools: a cross-sectional survey in London, UK

**DOI:** 10.1136/bmjopen-2016-011436

**Published:** 2017-01-06

**Authors:** Justin D Salciccioli, Dominic C Marshall, Mark Sykes, Alexander D Wood, Stephanie A Joppa, Madhurima Sinha, P Boon Lim

**Affiliations:** 1 Imperial College London, London, UK; 2 Department of Cardiology, Hammersmith Hospital, London UK

**Keywords:** cardiopulmonary resuscitation, automatic external defibrillator, education, cardiac arrest

## Abstract

**Objectives:**

Basic life support (BLS) training in schools is associated with improved outcomes from cardiac arrest. International consensus statements have recommended universal BLS training for school-aged children. The current practice of BLS training in London schools is unknown. The aim of this study was to assess current practices of BLS training in London secondary schools.

**Setting, population and outcomes:**

A prospective audit of BLS training in London secondary schools was conducted. Schools were contacted by email, and a subsequent telephone interview was conducted with staff familiar with local training practices. Response data were anonymised and captured electronically. Universal training was defined as any programme which delivers BLS training to all students in the school. Descriptive statistics were used to summarise the results.

**Results:**

A total of 65 schools completed the survey covering an estimated student population of 65 396 across 19 of 32 London boroughs. There were 5 (8%) schools that provide universal training programmes for students and an additional 31 (48%) offering training as part of an extracurricular programme or chosen module. An automated external defibrillator (AED) was available in 18 (28%) schools, unavailable in 40 (61%) and 7 (11%) reported their AED provision as unknown. The most common reasons for not having a universal BLS training programme are the requirement for additional class time (28%) and that funding is unavailable for such a programme (28%). There were 5 students who died from sudden cardiac arrest over the period of the past 10 years.

**Conclusions:**

BLS training rates in London secondary schools are low, and the majority of schools do not have an AED available in case of emergency. These data highlight an opportunity to improve BLS training and AEDs provision. Future studies should assess programmes which are cost-effective and do not require significant amounts of additional class time.

**Strengths and limitations of this study**

▪ Strengths include data collected prospectively from secondary schools across London, with responses reflecting practices as recent as October 2014.
▪ A number of schools, however, are not represented in the data set.
▪ Recall bias limits our ability to generalise our findings relating to the number school children who have died on school premises of sudden cardiac death.

## Introduction

Out-of-hospital cardiac arrest (OHCA) is a major public health burden which poses significant strain on healthcare systems^1^ with ∼420 000 arrests in the USA^2^ and 275 000 in Europe^3^ annually. Basic life support (BLS) training is associated with improved outcomes from cardiac arrest.^4^ Rates of bystander cardiopulmonary resuscitation (CPR) vary by location,^5^ a result which is thought to be the result of poor training and education in CPR practices or the result of poor implementation of training practices.

Advances in cardiac arrest care have, since it was first described in 1991, focused on improving performance in the chain of survival.^6^ While the past decade has significant improvement in the advanced care of the postarrest patient, multiple recent investigations have demonstrated in large populations of patients the overall importance of improving the early links in the chain of survival, namely early identification of cardiopulmonary arrest and early initiation of bystander CPR efforts via large-scale educational programmes.^7^ ^8^ Previous investigations have shown that BLS measures may be more important than advanced care in survival from OHCA,^9^ and more recent evidence has provided further support for this effect across healthcare systems.^10^ One area that remains unanswered is whether legislation to require schools to teach BLS will improve outcomes for patients suffering cardiac arrest.

In order to improve the rate of early bystander CPR and early defibrillation the International Liaison Committee on Resuscitation (ILCOR) recommended training in CPR and familiarisation with automated external defibrillator (AED) as part of secondary school curricula.^11^ BLS training has since become a requirement for graduation in multiple states throughout the USA.^12^ Recent efforts have been made globally to train children in CPR, and the WHO has endorsed the ‘Kids Save Lives’ statement from the European Resuscitation Council to further improve training for school-aged children.^13^ ^14^ However, within Europe variation exists between countries regarding the methods used to train and how much time is spent training children, the general public and healthcare professionals alike.^15–17^ The requirement for BLS training is not part of the educational curriculum in the UK, and the current practice of BLS training in schools throughout the UK is unknown.

The primary aim of this investigation was to appreciate the current practices of CPR and AED training in school-aged children in London. Our hypothesis was that, despite consensus statements from local and international professional resuscitation bodies, there would be a small proportion of London secondary schools currently offering universal BLS training programmes. This was based primarily on previous reports which have suggested low rates of BLS training in the UK compared to European countries.^15^ These data are relevant as they may highlight potential areas for improvement in public health initiatives. In order to identify training programmes, we performed a cross-sectional survey to ascertain current training practices for students in London secondary schools. To the best of our knowledge, this investigation is the only study to have assessed the rate of BLS training in London secondary schools since these consensus statements have been made.

## Methods

### Study design and setting

A registered audit was conducted as a cross-sectional survey of BLS and AED training in London secondary schools between June 2014 and October 2014. The project was approved as an audit at Hammersmith Hospital, Imperial College Healthcare Trust (Registration number 1673). A total of eight interviewers administered the survey during the course of two afternoon sessions. Prior to each session interviewers received via lecture presentation preliminary training in survey details and delivery for appropriate administration of the telephone interviews. All schools were contacted by email initially, and a subsequent telephone interview was conducted with school staff familiar with local training practices. Response data were anonymised and captured electronically in a standardised electronic database. In order to ensure standardisation of response data, the database contained prespecified variable responses as described below.

### Survey design and data collection

The survey was developed in two phases. First, a preliminary survey was created and school administrators in three prespecified London boroughs were contacted to complete the survey. Interviewers were responsible for recording survey results data as well as recording aspects of the survey tool which requiring additional clarification. Second, the survey tool was updated to incorporate suggestions from the initial series of telephone interviews and data collection. The final survey tool (see online supplementary files) was brief, included fewer than 20 response elements and could typically be completed over the telephone in <10 min.

10.1136/bmjopen-2016-011436.supp1 supplementary file

Response data were collected and managed using an electronic data capture tool which provided prespecified variable responses and missing value-response alerts to minimise incomplete responses. During the course of the telephone interview data were captured directly into the standardised web-based survey tool.

### Outcomes and analysis

The primary outcome of interest was a current universal training programme, which delivers BLS training to all students in the school. Secondary outcomes of interest included the presence of an AED in the school and the perceived barriers to implementation of a universal student training programme. The survey also included a single question to estimate the rate of death in school children during the 10-year period prior to the current investigation. Simple descriptive statistics including frequencies with percentages were used to summarise the results, as appropriate.

### Post hoc analysis

We performed a single post hoc analysis to estimate the cost to treat a single case of student cardiac arrest. During the course of the data collection period, the UK Department of Education (DoE) issued a public statement which recommends placement of AEDs in all UK primary and secondary schools.^18^ The DoE statement supports purchasing of AEDs by the UK National Health Service (NHS) Supply Chain and with this programme, the cost per AED would be £452.78.^18^ For this analysis, we used reported incidence of student sudden cardiac arrest as a single case of cardiac arrest and computed the rate of cardiac arrest per secondary school surveyed.

## Results

### Characteristics

**Figure 1:**
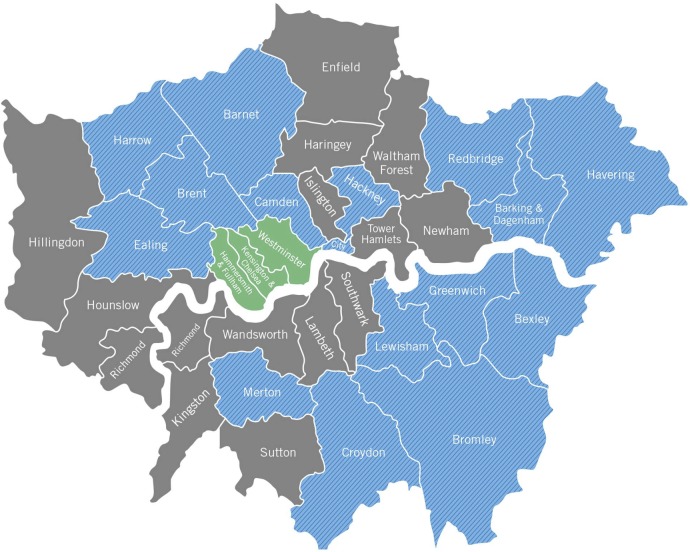
Map of London boroughs. Colours represent boroughs with surveys completed from all secondary schools within the borough (green), from a proportion but not all of the secondary schools (blue), or borough without completed survey data (grey).

Surveys were completed in 19 (59%) of the 32 London boroughs (figure 1). Of 449 schools, representatives from 71 (16%) schools were contacted successfully and a total of 65 (15%) completed the survey. There were 6 schools which refused to participate (8%). The student population from the surveyed schools completed covers an estimated population of 65 396 students between the ages of 11 and 19 years. In this sample, the earliest that students are exposed to BLS training is in year 7 (∼11 years of age). Specialist First Aid and CPR educators from outside organisations (eg, St. John's Ambulance, British Heart Foundation, etc) are most commonly responsible for providing educational programmes and training for students (15%).

### Primary and secondary outcomes

There were 5 (8%) schools that provide universal training programmes for students, and an additional 31 (48%) offered training as part of an extracurricular programme or chosen module. The most common reasons for not having a universal BLS training programme is the requirement for additional class time (28%) and that funding is unavailable for such a programme (28%). An AED was available in 18 (28%) schools, unavailable in 40 (61%) and 7 (11%) reported their AED provision as unknown. There were 5 students who died from sudden cardiac arrest over the period of the past 10 years. However, the administrator reporting 1 of the 5 cases was unable to confirm whether the sudden cardiac arrest happened at the school or away from school premises.

### Cost analysis

Of the 65 schools surveyed, there were at least 5 sudden cardiac arrests reported during the preceding 10-year period. As such, an AED would need to be placed in 13 secondary schools in order to provide the opportunity to treat at least 1 sudden cardiac arrest within a 10-year period. The NHS Supply Chain will provide AEDs in schools throughout the UK for £452.78, and for a total of 3628 state funded secondary schools this would amount to a cost of £1,642,685.84 in order to place one AED in every secondary school in England.^19^

## Discussion

In this prospective audit of London secondary schools, there were overall low rates of universal BLS training programmes for students. Specifically, fewer than half the schools surveyed offer some form of optional BLS training for students with only 8% offering a universal BLS training programme. The most common reasons stated for not having universal BLS training were the requirement for additional class time and the lack of funding to support such programmes. Further, we found less than a third of schools had an AED on the school premises available in case of emergency. Although small, the estimated number of students suffering sudden cardiac arrest while on school premises (5) in the preceding decade highlights the importance of current government and public health campaigns to equip schools with AEDs and to improve the rate of CPR training in schools. We estimate that an AED placed in a minimum of 13 schools would be required in order to treat a single case of a child with sudden cardiac arrest.

Our results demonstrate the significant room for improvement in student training in schools. We identified a small proportion (8%) of schools which provide CPR training for all students while 48% of schools offer some optional training for students. Our findings reflect the results of a similar survey of schools in Yorkshire, England, which found only 3 of the 14 schools delivered universal training.^20^ Our results are further in line with a recent investigation from Toronto, Canada, which found that 51% of secondary schools provide CPR training for students although it is unclear whether CPR training programmes were required for all students in the institution or whether these were optional programmes.^21^ There are important differences relating to the legislation between the two locations that raise the issue of making CPR training a requirement of school curriculum. While the government of Ontario, Canada, has made it mandatory for students to demonstrate an understanding of CPR in order to obtain their secondary school diploma, the UK government has yet to make such a change to curriculum requirements. It is unclear, therefore, what effect legislation has on the overall rate for students receiving CPR training. Despite this difference, one recent report has also shown a temporal trend towards improving outcomes from OHCA in Denmark since instituting mandatory resuscitation training programmes in elementary schools.^8^ A recent report from Denmark assessed which factors school leaders and teachers value in the aim to train school children in BLS.^22^ It is important to note that other investigations have demonstrated that effective BLS training is possible in the absence of trained or experienced BLS instructors.^23^ Further, while two randomised studies have demonstrated that trained teachers are able to provide adequate training in for resuscitation in schools, a systematic review has summarised the methods to best teach CPR to children.^24–26^

Our findings would suggest a rate of approximately one cardiac arrest per 130 school years (0.04 per 5 years), a similar rate to previous reports in the USA.^27^ On the basis of current evidence European Resuscitation Council (ERC) guidelines support the cost-effectiveness of placement of AEDs in public areas where there is one cardiac arrest per 5 years.^28^ Although below the threshold for cost-effectiveness recommended by the ERC, placement of an AED in schools may fall within the acceptable cost per Quality Adjusted Life Years (QALY) recommended by the National Institute of Clinical Excellence and will further increase awareness and familiarity with AEDs among school children. In the UK, where legislation for BLS training in schools has not been written, the Department of Education has recently published guidance for schools to equip the institution with an AED^18^ and offer financial support for purchasing an AED. As financial requirements were cited as one of the most common reasons for not having BLS training programmes, such additional financial support should help improve public access AEDs in schools. Other campaigns such as the ‘Defib in schools’ campaign, by the Arrhythmia Alliance (http://www.defibssavelives.org/defibs-in-schools), and the British Heart Foundation ‘Nation of Lifesavers’ community campaign to support AED installation in schools can help to increase public awareness and improve AED installation rates. Such campaigns are important especially in light of the fact that previous investigations have shown a significant improvement in neurologically intact survival with onsite AEDs compared to AEDs which have been dispatched via emergency services.^29^ In the absence of legislation for BLS and AED training, local champions have been able to significantly increase rates of BLS and AED training and such endeavours should be encouraged, with successful local frameworks for provision of training adopted and implemented nationally.

The strengths of this investigation are that data were collected prospectively from school administrators who were responsible with BLS training practices at each of the institutions. We used an electronic data capture tool with prespecified variable responses and error-response elements to minimise incomplete responses. A number of limitations should also be considered when interpreting the results of this investigation. While we were able to capture survey results data on a large number of London secondary schools, there are a number of schools which are not represented. We have however collected complete survey data from 65 secondary schools from 19 boroughs across London, which represents a large estimated student population with responses reflecting practices as recent as October 2014. One further limitation is that the survey tool that was used for the investigation has not been validated in a separate population. We attempted to mitigate this weakness with a pilot phase of the survey during which time respondents were able to provide feedback about the questionnaire. Finally, as we have attempted to obtain an estimate of the number of school children which have died as a result of cardiac arrest, these data are inevitably limited by recall bias and larger population studies would be necessary to confirm our findings.

## Conclusions

BLS training rates in London secondary schools are low, and the majority of schools do not have an AED available in case of emergency. As a number of international health and resuscitation organisations are attempting to improve overall training rates, these data highlight an opportunity to vastly improve training in schools throughout the UK.
