# Supplementary material for: Basic life support education in secondary schools: a cross-sectional survey in London, UK
Source: BMJ Open. 2017 Jan 6;7(1):e011436. doi: 10.1136/bmjopen-2016-011436 (PMC5223627; doi:10.1136/bmjopen-2016-011436)
Supplement: supplementary file [file bmjopen-2016-011436supp_appendix.pdf]

Title: Basic Life Support Education in Secondary Schools: a cross-sectional survey in London, United Kingdom

Supplementary Appendix

Table of Contents:

Survey Instrument ………………………………………………………………………… 2

Survey Instrument

1. Borough: drop down list
    a. Barking and Dagenham, Barnet, Brexley, Brent, Bromley, Camden, City of London, Croydon, Ealing, Enfield, Greenwich, Hackney, Hammersmith and Fulham, Haringey, Harrow, Havering, Hillingdon, Hounslow, Islington, Kensington and Chelsea, Kingston, Lambeth, Lewisham, Merton, Newham, Redbridge, Richmond on Thames, Southward, Sutton, Tower Hamlets, Waltham Forest, Wandsworth, Westminster
2. School ID
3. What is the current enrollment (number of pupils) in this school?
4. What is the maximum age and minimum age of pupils in this school? (Give range, e.g. 8-14)
5. Is there currently any universal CPR education or training program for students in place in this institution, i.e. will ALL students at some point receive? (Y/N)
    a. If yes, continue to Q6
    b. If no, continue to Q10
6. In which year(s) are students introduced universally to CPR and AED training? Provide earliest year in which students are exposed to CPR training.
    a. Year 7
    b. Year 8
    c. Year 9
    d. Year 10
    e. Year 11
    f. Year 12 (lower 6th)
    g. Year 13 (upper 6th)
    h. Other (specify)
7. Are students exposed to CPR training at any other time? If so, in which years? Choose one.
    a. No follow-up training program in place
    b. Year 7
    c. Year 8
    d. Year 9
    e. Year 10
    f. Year 12 (lower 6th)
    g. Year 13 (upper 6th)
    h. Other (specify)
8. Who is responsible for providing CPR training / education for these students? Choose one.
    a. Teachers
    b. Specialist first aid/CPR educators employed at the institution
    c. Specialist first aid/CPR educators from an outside organization (e.g. St. John's ambulance, British Heart Foundation, etc.)
    d. Other (specify)
9. What has been the biggest challenge to maintain universal CPR training in this institution? Choose one.

    a. Instructor training or scheduling
    b. Class scheduling
    c. Initial purchase of equipment
    d. Maintenance of equipment
    e. Other (specify)

10. Is there any non-universal CPR training program for students at this institution? (Y/N)
    a. If yes, continue to Q13
    b. If no, continue to Q11
11. For schools without current CPR training/education programs:
    a. What do you perceive to be the biggest barrier to student training? Choose one.
        i. Requirement for additional class time
        ii. Funding unavailable for these programs
        iii. Cost of purchasing and/or maintaining equipment is prohibitive
        iv. School population is too small to be cost effective
        v. School population is too large to offer training to everyone
        vi. No perceived need for this training
        vii. Other (specify)
12. Other CPR training programs
    a. Are teachers or staff members required to have CPR training (Y/N)
        i. If yes, is this training optional or a requirement? Choose one.p
            1. Required for all teachers in this institution
            2. Required for select teachers in this institution
            3. Other (specify)
    b. Who is responsible for providing CPR training/education for these individuals?
        i. Teachers
        ii. Specialist first aid/CPR educators employed at the institution
        iii. Specialist first aid/CPR educators from an outside organization (e.g. St Johns ambulance, British Heart Foundation, etc.)
        iv. Other (specify)
    c. With what frequency, if any, do these individuals complete their CPR training? Choose one.
        i. Annually
        ii. Every second year
        iii. Every third year
        iv. Less than once every third year
        v. Never
        vi. Other
13. Non-universal CPR training for Students
    a. How might students be exposed to CPR training at this institution? Choose one.
        i. As part of Duke in Edinburgh scheme
        ii. Physical education classes
        iii. Social health and wellness class

iv. Other scheduled class time
v. Other (specify)

b. Who is responsible for providing CPR training/education for these students? Choose one.
    1. Teachers
    2. Specialist First Aid / CPR educators employed at the institution
    3. Specialist First Aid / CPR educators from an outside organization (eg St. John's ambulance, British Heart Foundation, etc.)
    4. Other (specify)

14. Is there an automated external defibrillator in case of emergency? (Y/N/unknown)
15. To the best of your knowledge, in the past 10 years, have any students passed away from sudden cardiac arrest (SCA)? Y/N/unknown
    a. If yes, what number of students have passed away from SCA (Total # of students within the last 10 years)
16. We are planning to survey a number of secondary schools in order to better understand CPR training practices across London. Would you be interested in receiving a summary of the results of this survey in the coming months? (This question is optional as a response here may remove anonymity in the survey. We do not plan to use this information for any other purposes) Y/N
17. Are you interested in learning more about the possibility of having student volunteers into your school to teach CPR and First Aid?
18. Can you provide the best email address to contact you about the above? (Record this information on the hardcopy list of schools provided)
19. Date/Time questionnaire completed
    a. Month, day, year, hour, min, AM/PM
